# “Awake” extracorporeal membrane oxygenation (ECMO): pathophysiology, technical considerations, and clinical pioneering

**DOI:** 10.1186/s13054-016-1329-y

**Published:** 2016-06-30

**Authors:** Thomas Langer, Alessandro Santini, Nicola Bottino, Stefania Crotti, Andriy I. Batchinsky, Antonio Pesenti, Luciano Gattinoni

**Affiliations:** Dipartimento di Anestesia, Rianimazione ed Emergenza Urgenza, Fondazione IRCCS Ca’ Granda-Ospedale Maggiore Policlinico, Via F. Sforza 35, 20122 Milan, Italy; The Geneva Foundation, Tacoma, WA USA; Multi Organ Support and Preservation Task Area, U.S. Army Institute of Surgical Research (USAISR), Ft. Sam Houston, Texas USA; Dipartimento di Fisiopatologia Medico-Chirurgica e dei Trapianti, Università degli Studi di Milano, Milan, Italy

## Abstract

Venovenous extracorporeal membrane oxygenation (vv-ECMO) has been classically employed as a rescue therapy for patients with respiratory failure not treatable with conventional mechanical ventilation alone. In recent years, however, the timing of ECMO initiation has been readdressed and ECMO is often started earlier in the time course of respiratory failure. Furthermore, some centers are starting to use ECMO as a first line of treatment, i.e., as an alternative to invasive mechanical ventilation in awake, non-intubated, spontaneously breathing patients with respiratory failure (“awake” ECMO). There is a strong rationale for this type of respiratory support as it avoids several side effects related to sedation, intubation, and mechanical ventilation. However, the complexity of the patient–ECMO interactions, the difficulties related to respiratory monitoring, and the management of an awake patient on extracorporeal support together pose a major challenge for the intensive care unit staff. Here, we review the use of vv-ECMO in awake, spontaneously breathing patients with respiratory failure, highlighting the pros and cons of this approach, analyzing the pathophysiology of patient–ECMO interactions, detailing some of the technical aspects, and summarizing the initial clinical experience gained over the past years.

## Background

Venovenous extracorporeal membrane oxygenation (vv-ECMO) has been classically employed as a rescue therapy for patients with respiratory failure not treatable with conventional mechanical ventilation alone [1, 2]. In recent years, however, ECMO is often started earlier in the time course of the acute respiratory distress syndrome (ARDS) in order to avoid possible detrimental effects of mechanical ventilation, such as ventilator-induced lung injury [3–5]. It might be of interest to underline that, in the mechanically ventilated patient on ECMO, two lungs are contributing to respiration: the membrane lung, which is extremely efficient, and the native, failing lung, which can contribute only partially to gas exchange. For this reason, some centers are pursuing the idea of using ECMO as a first line treatment, i.e., as an alternative to invasive mechanical ventilation in awake, non-intubated, spontaneously breathing patients with respiratory failure (“awake” ECMO). On one hand, this type of approach has several advantages as it could avoid mechanical ventilation-associated side effects; on the other, of course, its application is associated with several challenges.

In the present review we summarize the current knowledge on and initial experience with the use of vv-ECMO in awake, spontaneously breathing patients with respiratory failure.

## Pros and cons of spontaneous breathing

Breathing is an active task in any surface-living animal with lungs and in all marine mammals. “*Spontaneous*” was an unnecessary adjective to the word “breathing” until the era of mechanical ventilation began. Since then, controversies have arisen regarding the advantages and disadvantages of maintaining spontaneous breathing in critically ill patients with respiratory failure [6, 7], whose treatment mainstay is still invasive mechanical ventilation.

In this section we summarize the possible advantages (*pros*) and disadvantages (*cons*) of spontaneous breathing.

### Pros

1. S*pontaneously breathing* patients preferentially move the dorsal, more compliant part of their diaphragm. Ventilation is therefore directed towards the most dependent and better perfused parts of the lung, leading to optimal ventilation–perfusion matching [7]. In contrast, the shape of the diaphragm is altered in the sedated, paralyzed, *mechanically ventilated* patient [8], leading to a preferential passive movement of the paralyzed diaphragm and ventilation of the *non-dependent* lung. This worsens ventilation–perfusion matching during mechanical ventilation (Fig. 1).

**Fig. 1:**
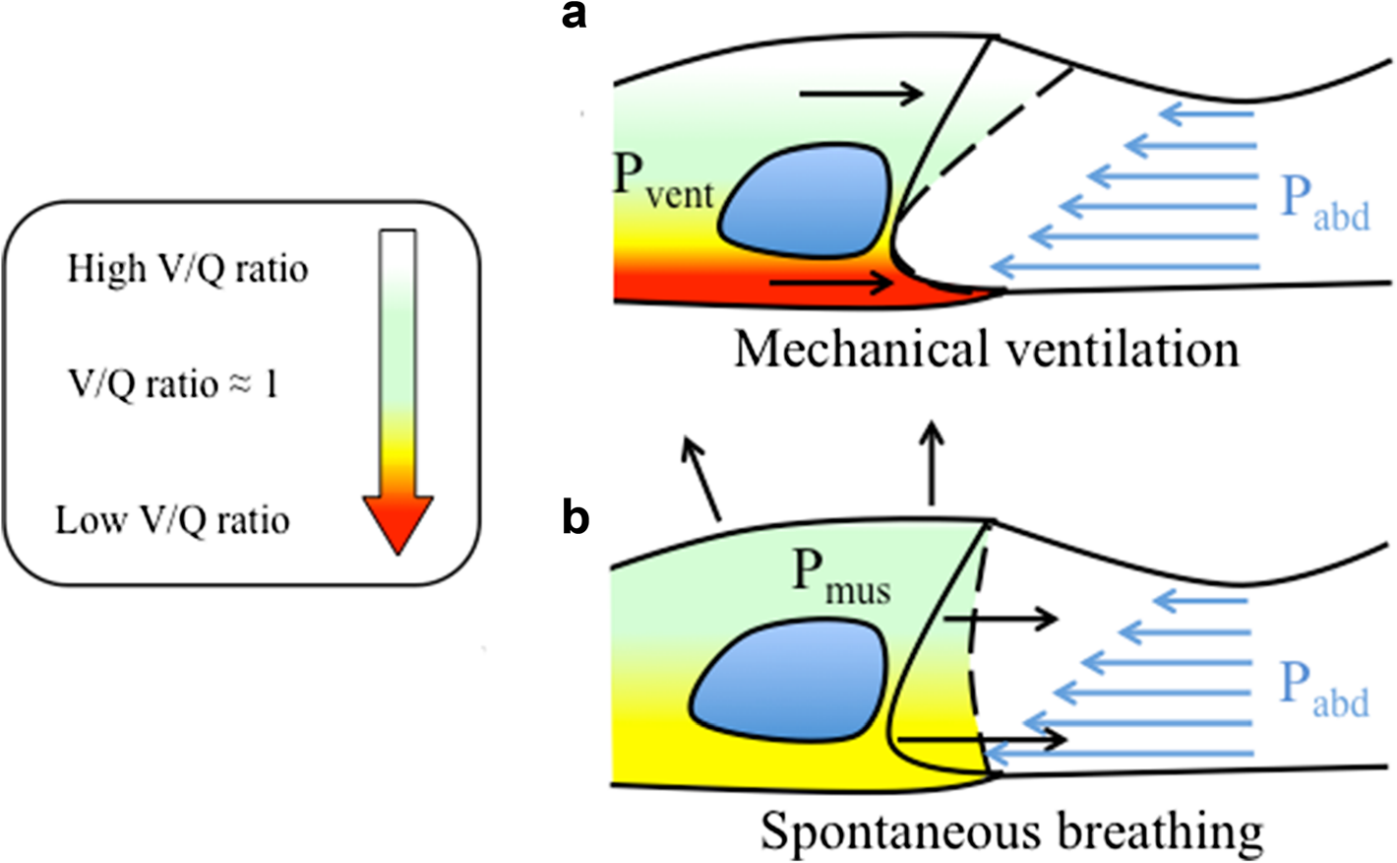
Diaphragm motion and ventilation/perfusion distribution in the awake and in the anesthetized subject. The lung ventilation-to-perfusion ratio (*V/Q*) is color-coded from *white* (high V/Q), to *green* (V/Q ≈ 1), to *red* (low V/Q). Diaphragm shape at end expiration (*continuous line*) and end inspiration (*dashed line*) in the supine position is shown. Intra-abdominal pressure increases in the ventro-dorsal direction due to gravity (*blue arrows*) and displaces the dorsal part of the diaphragm more cephalad than the ventral part at end expiration. During mechanical ventilation the pressure applied by the mechanical ventilator displaces the ventral part of the diaphragm, which faces less intra-abdominal pressure, more than the dorsal part (passive movement). Ventilation will thus be distributed preferentially to the ventral lung regions, increasing the ventilation-to-perfusion ratio (*V/Q*) of these areas. In contrast, dorsal lung regions will receive less ventilation and their V/Q will be lower (**a**). During spontaneous breathing (either assisted or unassisted), both the ventral and the dorsal part of the diaphragm move (active contraction). Ventilation will distribute more homogeneously along the ventro-dorsal axis of the lung and will more closely match perfusion (V/Q ≈ 1) (**b**)
2. The tone of the respiratory muscles in the awake, spontaneously breathing subject guarantees the expansion of the chest wall and lungs at end expiration (functional residual capacity). In contrast, anesthesia (with or without paralysis) leads to loss of muscle tone, inward displacement of the ribcage [9], and decreased functional residual capacity [10], which, in turn, can favor the formation of atelectasis. Of note, this mechanism is more pronounced in edematous, “heavy”, ARDS lungs [11].
3. Maintaining diaphragmatic contraction and avoiding controlled mechanical ventilation could prevent ventilator-induced diaphragm dysfunction [12].
4. During spontaneous breathing, air moves from the mouth to the alveoli through a decrease in intrathoracic pressure, which favors venous return from extra-thoracic organs, maintaining cardiac filling and output. The same mechanism seems implicated in favoring pulmonary lymphatic drainage [13]. On the other hand, with mechanical ventilation the intrathoracic pressure increases during inspiration, having the opposite effect of reducing venous return, cardiac output, and thoracic lymph flow [13, 14].
5. The avoidance of endotracheal intubation could reduce the incidence of ventilator/intubation-associated pneumonia through the maintenance of the natural barrier defenses against bacteria [15].

### Cons

1. Transpulmonary pressure is one of the forces implicated in the development of ventilator-induced lung injury [16–18]. Spontaneous breathing generates positive transpulmonary pressure (airway pressure minus pleural pressure) similarly to mechanical ventilation. Lung damage might therefore also derive from spontaneous hyperventilation (spontaneous ventilation-induced lung injury) [19–22].
2. When the work of breathing is very high, the strenuous respiratory muscle effort can lead to high oxygen (O_2_) consumption (and carbon dioxide (CO_2_) production), i.e., a high cost of breathing, which in turn could worsen hypoxemia. If gas exchange needs are not met in other ways (e.g., through extracorporeal respiratory support), sedation, intubation, and mechanical ventilation could be necessary to avoid muscle exhaustion.
3. Emergent intubation and initiation of mechanical ventilation might become necessary in the awake, spontaneously breathing, non-intubated patient in cases of ECMO equipment failure.

## Pros and cons of keeping patients awake

Managing awake patients is a relatively new and challenging task in medical intensive care units (ICU).

### Pros

1. *Reduction of delirium*: the pathogenesis of delirium in the ICU is multifactorial, one of the main determinants being the use of sedative drugs [23]. Avoiding or reducing the amount of hypnotic agents could therefore reduce the development of this disturbance, which is associated with prolonged ICU/hospital stay and mortality [24].
2. *Rehabilitation*: muscle mass loss and critical illness myopathy and polyneuropathy often affect ICU patients [25]. Awake patients can actively collaborate with physiotherapists to perform physical rehabilitation, therefore reducing the incidence of these neuromuscular disorders [26]. The organizational effort needed to safely perform physical therapy in ECMO patients needs to be considered. In our experience the multidisciplinary team includes two physical therapists, two nurses, and a physician.
3. *Interaction with relatives/friends and medical staff*: patient–relative interactions comprise one of the most striking differences between the ICU and other hospital wards. The ability of an awake patient to communicate with friends and relatives could render the unfriendly ICU environment a more usual and easy to cope with situation for both patient and visitors. Furthermore, it is possible for an awake patient to communicate her/his symptoms and needs to the medical staff, something that is heavily underappreciated and yet is a fundamental source of information about the patient’s condition and response to therapy.

### Cons

1. *Risk of invasive device displacement*: awake patients must be carefully monitored and instructed not to remove any invasive device to avoid the risk of self-harm. This is of particular relevance for patients on ECMO.
2. *Patient discomfort, pain and anxiety*: an awake patient requires analgesics for invasive device tolerance and pain control (e.g., movements during physiotherapy, invasive procedures). Furthermore, the ICU can be a very stressful environment because of the procedures/actions undertaken on the awake patient as well as those on other patients. Depending on the organization of the ICU (single room versus open-plan), supplemental care is required with regard to what is said and done in the proximity of an awake patient.

## Physiology and pathophysiology of the control of breathing

In normal physiology, spontaneous breathing is largely controlled by the PCO_2_ (partial pressure of carbon dioxide; and therefore pH) and O_2_. On one hand, the hypoxic ventilatory response is activated only at low levels of PO_2_ (partial pressure of oxygen; 40–50 mmHg) and is, therefore, rarely the primary respiratory drive [27]. On the other hand, the key role of CO_2_ as a trigger for respiration has been clearly identified, including through experiments performed with the use of extracorporeal gas exchange [28, 29]. Our knowledge on the control of breathing is, however, largely limited to studies performed on healthy animals and humans. In conditions of acute lung disease, lung receptors that are silent in normal physiological conditions might be activated and could play a role in determining the respiratory pattern of these patients [30]. Interestingly, typical features of lung disease, such as systemic and local pulmonary inflammation [29, 31–33], lung collapse, and lung microembolism [34], have been shown to activate these receptors.

## Physiology and pathophysiology of spontaneous breathing

Breathing is a complex function that has the ultimate purpose of delivering oxygen to every cell of the organism in order to perform cell respiration, i.e., the production of energy from the oxidation of a substrate in the presence of an acceptor of electrons—oxygen. The other purpose of breathing is the clearance of the waste product of cellular respiration, namely carbon dioxide.

Schematically, the respiratory system is composed of a pump—the respiratory muscles—and a gas exchanger—the lungs. The pump provides the force needed to expand the ribcage and lungs, while the coupling between these two structures is provided by the pleura. During spontaneous inspiration the ribcage expands and the diaphragm is displaced in the caudal direction. The resultant decrease in pleural pressure decreases the alveolar pressure to subatmospheric levels and, due to this pressure gradient, air reaches the alveoli through the tracheo-bronchial tree. Here, gases are passively transferred to and from blood according to their partial pressure gradients.

The simplified equation of motion (Eq. 1) describes the force that must be exerted by the respiratory muscles in order to move air from the atmosphere to the lungs:

1 $$ {\mathrm{P}}_{\mathrm{mus}} = \mathrm{V}\ *\ \mathrm{E} + \overset{.}{\mathrm{V}}\ *\ \mathrm{R} $$

where P_mus_ represents the pressure exerted by the respiratory muscles, E and R the respiratory system elastance and resistance, respectively, and V and V̇ the volume and flow, respectively, of gas entering the respiratory system.

During the healthy state, increases in respiratory muscular effort are usually due to increased CO_2_ production, e.g., during exercise, with consequently increased ventilation (V and V̇) at fairly constant mechanical characteristics of the lung (E and R). In patients with acute lung injury, E is typically worsened due to the accumulation of edema, which causes a reduction in ventilatable lung volume [11]. Furthermore, airway hyperreactivity and inflammation may lead to an increase in R in ARDS patients [35]. Finally, due to the presence of a high dead space fraction [36], an increased minute ventilation is needed in order to remove the metabolically produced CO_2_. The combination of these factors results in the need for greatly increased muscular effort (P_mus_) to satisfy the gas exchange needs. Of note, the increased work of breathing increases oxygen consumption and CO_2_ production by the respiratory muscles, potentially leading to a vicious cycle which accelerates the process of muscular exhaustion.

Available therapeutic options operate on very different concepts. On the one hand, therapy aims at improving lung function (E and R) through different pharmacological interventions, e.g., inhaled bronchodilators or antibiotics. On the other, time is needed for such therapy to be effective and, therefore, supportive therapy of the failing respiratory system is required. During controlled mechanical ventilation the pressure generated by the ventilator (P_vent_) replaces P_mus_, therefore acting on the left side of Eq. 1. This type of intervention has a straightforward application during respiratory failure due to neuromuscular diseases, such as amyotrophic lateral sclerosis, poliomyelitis, and others, or during general anesthesia where muscular function is pharmacologically abolished.

In contrast, vv-ECMO acts on the right side of Eq. 1, lowering the need to breath (V and V̇), by replacing the gas exchange function of the lung (CO_2_ removal and blood oxygenation). Of note, this type of intervention would be the logical approach in cases of respiratory failure due to lung disease, i.e., conditions in which the respiratory muscles are unaffected by the disease and the lung function is impaired.

## Technical considerations

### Cannulation approaches

Venous cannulation for vv-ECMO is preferentially performed percutaneously using the Seldinger technique [37] and may involve two different approaches: (i) single site cannulation using bicaval dual-lumen catheters; or (ii) dual site cannulation (femoro-jugular or femoro-femoral). Advantages of single site, bicaval dual-lumen cannulae include freeing up the femoral veins, thus favoring passive and active physical therapy [38], and reduced risk of catheter-related infection and insertion site bleeding [39]. The catheters used in adults range between 27 and 31 Fr (9–10.3 mm diameter), i.e., substantially larger diameters are needed compared with dual site catheters (between 19 and 25 Fr) in order to guarantee sufficient blood flows through the membrane lung [40]. Positioning bicaval dual-lumen catheters is a major challenge and fluoroscopy and/or transesophageal echocardiography are usually needed to guarantee the correct placement [41–43]. Of note, both ventricular rupture during placement and displacement of the cannula into the right ventricle or hepatic veins during ECMO support, with consequent inadequate ECMO blood flow and reduced respiratory support, have been described [44, 45]. As cannula displacement could be related to activity/agitation of patients, the use of bicaval dual-lumen cannulae should be done with caution in potentially agitated patients or patients with extremely severe respiratory failure. Due to the complexity and associated risks, patients are usually anesthetized and intubated for the placement of bicaval dual-lumen cannulae. In contrast, when using the dual site approach, cannulation may also be performed in awake, spontaneously breathing patients under light sedation and local anesthesia. However, at least one cannula needs to be placed at the groin, i.e., in the femoral vein, resulting in limited possibility to perform physical therapy.

### Physiology of extracorporeal gas exchange and patient–machine interactions

The physiological rules regulating pulmonary gas exchange also apply to extracorporeal gas exchange. Indeed, *CO*_*2*_*removal* depends primarily on *ventilation* of the membrane lung, i.e., on sweep gas flow, and, to a lesser extent, on the PCO_2_ of blood entering the ECMO system. Furthermore, CO_2_ removal depends, in a logarithmic relationship, on the blood flow through the membrane lung [28].

The major determinants of oxygen transfer are the extracorporeal blood flow and the hemoglobin saturation of blood entering the membrane lung [46], given that blood oxygenation is a saturable process. Of note, metabolically produced CO_2_ (approximately 200 ml/min) can, in theory, be cleared from a low quantity of venous blood (0.5–1 L), while significantly higher blood volumes are required in order to provide an equivalent amount of oxygen.

**Fig. 2:**
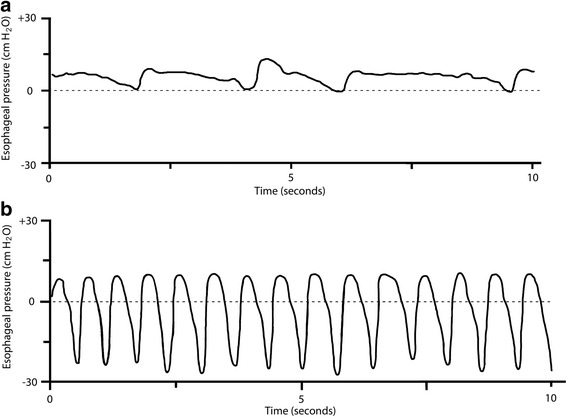
Esophageal pressure swings during spontaneous breathing in normal conditions and with ARDS. **a** Esophageal pressure (P_es_) trace of an awake, spontaneously breathing sheep with healthy lungs. Esophageal pressure swings (∆P_es_) are around 4–6 cmH_2_O and the respiratory rate is around 14–18 breaths per minute. **b** P_es_ trace of an awake, spontaneously breathing sheep with oleic acid-induced ARDS. Measured ∆P_es_ values are around 20–30 cmH_2_O and respiratory rate is greatly increased. Personal experimental data of Thomas Langer and Andriy Batchinsky [49]

**Fig. 3:**
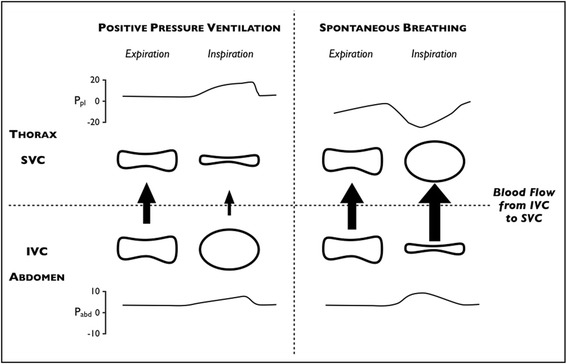
Shape of the intra- and extrathoracic veins in different transmural pressure conditions (heart–lung interactions). Changes in pleural pressure (*P*_*pl*_), abdominal pressure (*P*_*abd*_), the shape of the superior and inferior venae cavae (*SVC* and *IVC*, respectively), and the amount of blood flow (*arrows*) from the abdomen to the thorax during mechanical ventilation (*left panel*) and spontaneous breathing (*right panel*). During positive pressure ventilation, the increased pleural pressure squeezes the SVC and reduces blood flow from the abdominal compartment. This induces a distention of the IVC, favoring blood drainage to the extracorporeal circuit. During spontaneous breathing with high inspiratory effort, the significant decrease in pleural pressure dilates the SVC and increases blood flow from the abdominal compartment. This may induce a collapse of the IVC, hindering blood drainage to the extracorporeal circuit

The awake, spontaneously breathing patient is an independent and unpredictable variable with potentially relevant implications for patient–machine interactions. Indeed, the maximum blood flow through the ECMO system depends on not only the size of the cannula, as mentioned above, but also the adequacy of the central volume status, i.e., preload and venous return. The volume status is influenced by heart–lung interactions and, in spontaneously breathing patients, particularly by the periodic negative intrathoracic pressures caused by the activity of the respiratory muscles. During normal, physiological spontaneous breathing, pleural pressure swings are small, around 4–6 cmH_2_O [47] and the hemodynamic effect is usually negligible. Nevertheless, during respiratory distress, pleural pressure swings can increase significantly (often reaching 20–30 cmH_2_O) and diaphragmatic excursions can be very pronounced, even during extracorporeal support (Fig. 2). This, in turn, can lead to a blood shift from the inferior to the superior vena cava and to a collapse of the inferior vena cava caused by increased abdominal pressure. The net result, in the case of blood drainage from the inferior vena cava, could be the collapse of the vein around the drainage cannula with subsequent reduction in blood flow (Fig. 3). Of note, bicaval dual-lumen catheters, which drain from both the intra- and extra-thoracic compartments, should be less affected by this kind of interaction.

Also, blood reinfusion (afterload) can become critical in the case of increased intrathoracic pressure, such as during coughing or Valsalva’s maneuver, leading to relevant, though usually transient, reductions in blood flow.

In addition to mechanical patient–machine interactions linked to blood drainage and reinfusion, physiological and metabolic interactions caused by extracorporeal gas exchange also take place. Extracorporeal CO_2_ removal, the primary and most efficient effect of ECMO support, allows respiratory acidosis to be corrected when present, thus potentially reducing pulmonary vascular resistance and improving systemic hemodynamics [48]. The respiratory response to CO_2_ removal, initially observed in experimental studies, is to decrease minute ventilation, up to apnea, when 100 % of the metabolically produced CO_2_ is removed by the membrane lung [28, 49, 50]. This will reduce the work of the respiratory muscles, lowering the cost of breathing, which may be as high as 50 % of total oxygen consumption in patients with respiratory failure [49, 51].

In clinics, it has been anecdotally observed that some ECMO patients respond physiologically, i.e., decrease their alveolar ventilation proportionally to the amount of CO_2_ removed extracorporeally, while others tend to vary their ventilation only slightly with variations of CO_2_removal (manuscript submitted). Reasons for such differences are under investigation but hypotheses include the effect of agitation, discomfort, and cough and the involvement of mechanisms other than pH/PCO_2_/PO_2_ in the control of breathing and dyspnea generation, e.g., activation of pulmonary receptors [30, 32, 52].

Of note, hypoventilation induced by extracorporeal CO_2_ removal will lower the global ventilation-to-perfusion ratio of the natural lung and could lead to reabsorption atelectasis, therefore worsening hypoxemia [53]. To avoid atelectasis it is useful to (i) titrate sweep gas flow in order to relieve dyspnea and avoid high pleural pressure swings, (ii) maintain a certain degree of spontaneous respiratory activity to avoid hypoventilation-related atelectasis, and (iii) increase mean airway pressure through the application of continuous positive airway pressure (CPAP) or non-invasive ventilation.

Increasing the supply of oxygen through the membrane lung, thus increasing arterial PO_2_, has a smaller effect on ventilation compared with CO_2_ removal, as the hypoxic respiratory drive is usually involved only at low PO_2_ values [27]. Nevertheless, it was demonstrated that increasing the inspiratory fraction of oxygen and therefore PO_2_ in hypoxic ARDS patients on pressure support ventilation (leaving all other parameters unchanged) caused a reduction in minute ventilation, mainly attributable to a reduced respiratory rate [54].

Finally, vv-ECMO increases venous oxygen saturation and venous oxygen tension and could, therefore, reduce hypoxic pulmonary vasoconstriction, having a twofold effect, (i) increasing the shunt fraction and (ii) reducing pulmonary arterial pressure, therefore indirectly unloading the right ventricle [55, 56].

### Monitoring during awake ECMO

Hemodynamic monitoring in awake, spontaneously breathing patients on ECMO does not differ from the monitoring performed in sedated and intubated ECMO patients. Conversely, respiratory monitoring is a major challenge. Indeed, physicians are blinded to both airway pressures and tidal volumes, which are the mainstays of respiratory monitoring in ventilated patients.

Physicians need, therefore, to rely on the clinical evaluation of signs and symptoms of respiratory distress, such as respiratory rate, dyspnea, rapid shallow breathing, and others. In addition, some centers monitor esophageal pressure swings, i.e., a surrogate of variations in pleural pressure generated by the inspiratory muscles [57]. These pressure swings are evaluated in dynamic conditions and represent, therefore, the pressure applied to the alveoli to overcome both the elastic and resistive workload. The elastic component is the transpulmonary pressure producing alveolar inflation, while the resistive component generates flow through the airways. Of note, both components, i.e., transpulmonary pressures and negative inspiratory pressures due to resistive workload, could, in the case of very high values, worsen the underlying lung injury [58]. Therefore, from our point of view, and regardless of the cause of the increased esophageal swings, these need to be controlled in order to avoid additional pulmonary injury. Usually, reduction of high esophageal swings can be achieved by increasing the sweep gas flow, therefore increasing extracorporeal CO_2_ removal. If not sufficient, light sedation could help to slightly reduce the respiratory drive. At our institution, despite the lack of sound clinical evidence on the topic, if a patient’s esophageal swings remain “dangerously” high (>15 cmH_2_O), patients are usually deeply sedated and switched to conventional invasive mechanical ventilation.

### Evaluation of gas exchange in the native lung

Assessment of gas exchange in the native, diseased lung is extremely difficult in patients on vv-ECMO. Proper evaluation of the *oxygenation* capabilities of the native lung is hindered by the increased shunt fraction caused by the partial loss of pulmonary hypoxic vasoconstriction.

On the other side, evaluation of the *CO*_*2*_*clearing* capabilities of the native lung is also challenging. At steady state, CO_2_ elimination (VCO_2_) equals CO_2_ production. During extracorporeal CO_2_ removal total VCO_2_ equals extracorporeal (V_M_CO_2_) plus patient CO_2_ elimination (V_L_CO_2_). V_M_CO_2_ can be easily measured by analyzing the composition of gas exiting the membrane lung and multiplying the CO_2_ concentration by the sweep gas flow. In contrast, measurement of V_L_CO_2_ is difficult in non-intubated patients. Side-stream capnographs measuring end-tidal CO_2_ in spontaneously breathing patients are now available and allow estimation of the alveolar dead space fraction; however, they do not provide volumetric data and V_L_CO_2_ cannot, therefore, be assessed.

Of note, the contribution of the membrane lung to gas exchange can be temporarily zeroed by interruption of the sweep gas (flow = 0 L/min) so that the performance of the native lung can be better evaluated. Nevertheless, this procedure is usually performed during evaluation of weaning from ECMO and rarely in patients requiring elevated extracorporeal support.

## Clinical pioneering

### Bridge to lung transplantation

The use of vv-ECMO in awake, non-intubated spontaneously breathing patients was first described for respiratory deterioration in patients awaiting lung transplantation (as a “bridge” to lung transplantation). These patients are the ideal candidates for an awake ECMO approach given their usual single organ dysfunction, their fragile heart–lung equilibrium, and the potential great benefit from the maintenance of preoperative physical rehabilitation [59, 60]. Several case series have been published over the past years (Table 1) and, more recently, a retrospective analysis with historical controls showed a better survival in awake ECMO patients compared with those on mechanical ventilation [61].

**Table 1:** Studies on awake ECMO as a bridge to lung transplantation

Reference	Year	Number of patients	Average bridge duration (days)	Type of extracorporeal support	Successful bridge
Olsson et al. [72]	2010	5	21	VA	4/5
Fuehner et al. [61]	2012	26	9	VV, VA	NA
Javidfar et al. [73]	2012	6	NA	VV, VA	NA
Hoopes et al. [74]	2013	18	11	VV (10), VA (2)	18/18
				PA-LA (2), RA-Ao (4)	
Crotti et al. [62]	2013	10	28	VV (8), VA (1), AV (1)	8/10
Lang et al. [75]	2014	5	21	AV (2), VV (2)	5/5
Mohite et al. [76]	2015	7	89	VV, VA	NA
Inci et al. [77]	2015	6	NA	NA	6/6

Studies on awake ECMO as a bridge to lung transplantation reporting at least five patients are presented in chronological order of publication. “Successful bridge” defines the number of patients bridged with “awake ECMO” to lung transplantation without the need for intubation and mechanical ventilation

*AV* arterio-venous, *NA* not available, *PA-LA* pulmonary artery-left atrium, *RA-Ao* right atrium-ascending aorta, *VA* veno-arterial, *VV* veno-venous

Furthermore, a recent study showed that patients bridged with awake ECMO spent less post-operative time on mechanical ventilation and had shorter ICU and hospital lengths of stay compared with patients bridged with mechanical ventilation [62]. Nonetheless, patients who experience a rapid respiratory deterioration, and therefore need preoperative invasive respiratory support (mechanical ventilation and/or ECMO), apparently have a higher postoperative risk of death compared with patients not requiring preoperative invasive respiratory support [63]. These findings suggest that the condition of preoperative patients has a higher impact on postoperative outcome than the type of respiratory support.

### Exacerbation of chronic obstructive pulmonary disease

Carbon dioxide retention, caused by dynamic hyperinflation, ventilation/perfusion mismatch, and reduction of alveolar ventilation, is a typical feature of acute exacerbation of chronic obstructive pulmonary disease (COPD). In the case of failed medical therapy and non-invasive ventilation, intubation and invasive mechanical ventilation might become necessary [64], exposing these patients to several mechanical ventilation-associated side effects.

Since COPD patients are usually characterized by hypercapnia and only mild hypoxemia, low blood flow extracorporeal CO_2_ removal systems could be sufficient to unload their respiratory system. Pioneering studies on the topic described a reduced intubation rate in acute exacerbations of COPD patients failing non-invasive ventilation and supported with extracorporeal gas exchange (Table 2) [65–68]. Furthermore, some authors described the possibility, through the use of extracorporeal CO_2_ removal, to facilitate extubation and perform physical therapy in COPD patients already supported with mechanical ventilation [65, 69]. Despite the lack of sound clinical evidence, this strategy seems to be promising given the known drawbacks of mechanical ventilation in this category of patients.

**Table 2:** Studies on awake ECMO in COPD and ARDS patients

Reference	Year	Type of disease	Number of patients	Average support duration (days)	Type of extracorporeal support	Successful management without IMV	Successful weaning from IMV
Kluge et al. [68]	2012	COPD	14	9	PECLA	13/14	NA
Burki et al. [66]	2013	COPD	20	4	Low flow ECCO_2_R	9/9^*^	3/11^§^
Abrams et al. [65]	2013	COPD	5	8	Low flow ECCO_2_R	NA	5
Del Sorbo et al. [67]	2015	COPD	25	2	Low flow ECCO_2_R	22/25	NA
Hoeper et al. [70]	2013	ARDS	6	10	VV	3/6	NA

Studies on awake ECMO for acute exacerbation of COPD or ARDS reporting at least five patients are presented in chronological order of publication and according to type of disease. “Successful management without IMV” defines the number of patients managed without invasive mechanical ventilation during the ICU stay. “Successful weaning from IMV” defines the number of patients already intubated, mechanically ventilated, and on ECMO who were weaned from invasive mechanical ventilation, extubated, and managed with awake ECMO. ^*^Patients of groups 1 and 2 and ^§^patients of group 3 of the original publication by Burki et al.

*ARDS* acute respiratory distress syndrome, *COPD* chronic obstructive pulmonary disease, *ECCO*_*2*_ *R* extra-corporeal CO_2_ removal, *IMV* invasive mechanical ventilation, *NA* not available, *PECLA* pumpless extra-corporal lung assist; *VV* veno-venous

### Acute respiratory distress syndrome

Recently, some groups have explored the possibility of using ECMO as an alternative to mechanical ventilation also in awake, spontaneously breathing ARDS patients [70, 71]. Available data are very scarce; nevertheless, patients of this sort with respiratory failure appear to be more complicated to treat with an awake ECMO approach given their frequent multiple organ dysfunction.

## Conclusions

There is a strong rationale for the use of vv-ECMO in the treatment of respiratory failure in awake, spontaneously breathing patients (awake ECMO) as it allows several side effects related to sedation, intubation, and mechanical ventilation to be avoided. However, the complexity of the heart–lung–ECMO system interactions and the difficulties related to respiratory monitoring and the management of an awake patient on extracorporeal support make the awake ECMO patient a major challenge for the ICU staff. At the time of writing, sound clinical data on the topic are scarce and future clinical studies are needed in order to improve our understanding of the pathophysiology of awake ECMO patients and to evaluate any possible outcome benefit compared with the current standard of care.

## Abbreviations

ARDS: acute respiratory distress syndrome
COPD: chronic obstructive pulmonary disease
E: respiratory system elastance
ECMO: extracorporeal membrane oxygenation
ICU: intensive care unit
PCO_2_: partial pressure of carbon dioxide
P_mus_: pressure generated by the respiratory muscles
PO_2_: partial pressure of oxygen
P_vent_: pressure generated by the mechanical ventilator
R: respiratory system resistance
V: volume of gas entering the respiratory system
V̇: flow air gas entering the respiratory system
VCO_2_: total carbon dioxide elimination
V_L_CO_2_: carbon dioxide elimination through the native lung
V_M_CO_2_: carbon dioxide elimination through the membrane lung
vv-ECMO: venovenous extracorporeal membrane oxygenation
